# High-Temperature Behaviour of Zn-Based Galvannealed Coatings on Steel

**DOI:** 10.3390/ma16093341

**Published:** 2023-04-24

**Authors:** Peter Gogola, Zuzana Gabalcová, Martin Kusý, Jana Ptačinová

**Affiliations:** Institute of Materials Science, Faculty of Materials Science and Technology in Trnava, Slovak University of Technology in Bratislava, Ulica Jána Bottu 25, 917 24 Trnava, Slovakia; zuzana.gabalcova@stuba.sk (Z.G.); martin.kusy@stuba.sk (M.K.); jana.ptacinova@stuba.sk (J.P.)

**Keywords:** hot-dip galvanising, Fe-Zn intermetallics, galvannealing, high-carbon steel, heat treatment, quenching, solid solution, phase evolution

## Abstract

The potential of using a Zn-based, hot-dip coating to limit steel scale formation was investigated. The phase evolution within a pure Zn and a Zn0.1Al coating on a medium-carbon (0.5 wt.% C, 0.25 wt.% Si) steel sheet during a series of heat treatment steps was investigated. Such Zn-based coatings react with the steel substrate depending on the actual heat treatment condition. A series of expected intermetallic phases was observed via SEM/EDX and XRD techniques, such as ζ, δ and Γ phases along the η(Zn) phase. The η(Zn) phase was transformed to mainly δ and Γ phases during galvannealing (500 °C). The rapid quenching from 850 °C enabled the formation of the supersaturated α-(Fe) solid solution with increased Zn content. A continuous, intact, ~20 µm thick coating was observed after the final step of the heat treatment procedure, while signs of liquid metal embrittlement (LME) were not observed near the coating/steel interface. This will ensure reliable protection against heavy scale formation on heat-treated steel parts.

## 1. Introduction

Steel parts are regularly subject to various forms of heat treatment. Under suboptimal conditions, these procedures can cause extensive oxide scale formation on the surface of the processed parts. This is especially true for the heat treatment of bulk pieces, where it can be challenging to ensure the necessary inert or reductive atmosphere. A suitable solution is to use coated half products in these processes. A galvannealed (GA) coating can minimise the unwanted surface oxidation during various heat treatment procedures. Depending on the type and temperature of the heat treatment step, many of the coating’s properties will be impaired. Nevertheless, as long as it remains intact, this limits unwanted scale formation. This approach is often used during hot stamping of low-carbon steels, where the half-product is often exposed to temperatures between 600 and 900 °C. Wang et al. [1] investigated multiple heat exposure conditions expected during the hot-stamping process. Over 750 °C mainly super-saturated α(Fe) solid solution was observed in the coating. Very similar conclusions were reached by Hwang et al. [2] and Lee et al. [3]. At the same time, signs of liquid metal embrittlement (LME) must not be observed on the interface between the coating and the substrate. As summarised by Chakraborty [4], this relates to multiple processes using elevated temperatures where Fe-Zn phases can exist in a semi-molten state [5]. Wang et al. [1] and Hwang et al. [2] studied this issue more closely, specifically during hot-stamping of GA-coated steel parts.

The above-mentioned temperature range (600–900 °C) suggests that this approach can also be applied for other types of heat-treatment processes. Thus, a similar approach should also be applicable when processing steel grades for quenching and tempering.

In the current research, the feasibility of such an approach was investigated for a steel grade with increased carbon content. Usually, GA-coated half-product is processed. However, to better understand the behaviour of the coating, we chose to start from an uncoated steel substrate. The phases formed by the diffusion reaction between the Zn-based coating and the steel substrate during individual processing steps were followed up.

After galvanising in pure Zn, a well-known coating structure with η(Zn), ζ, δ and Γ phases is expected to form [6,7,8,9]. Gradual addition of Al in the range of 0.1–0.2 wt.% changes the formation of these phases significantly by forming an Fe-Al-based inhibition layer, usually reported as Fe_2_Al_5-x_-Zn_x_ [6,10,11,12,13,14,15]. While, generally, a continuous inhibition layer is reported to form above 0.15 wt.%Al in the bath, several publications indicate that even lower Al content has an influence on the formation of the Fe-Zn alloy layer. As reported by Baril et al. [14] previously, 0.10 to 0.13 wt.% Al coatings exhibit the presence of at least a discontinuous Fe-Al-based inhibition layer. The studies of Price et al. [13] report that such layers can be formed by FeAl_2_-Zn_x_ phase particles. On the other hand, based on FIB/TEM analysis, Zapico-Álvarez et al. [15] have stated that even such an incomplete inhibition layer is formed by the Fe_2_Al_5_-Zn_x_ phase and is followed by a δ phase layer. For a 0.13 wt.% Al bath, Fe_2_Al_5_-Zn_x_ and FeAl_3_ phases have been reported to form the inhibition layer by Chen et al. [10].

Galvannealing at a temperature of 500 °C supports the formation of ζ, δ and Γ phases at the expense of the η(Zn) layer [2,3,13,16]. Exposing such a GA-coated steel to the austenitising temperature leads to the formation of an α(Fe) solid solution with increased Zn content. As a consequence of the subsequent fast cooling (several tens of °C/s), this phase remains present even at room temperature in the form of a supersaturated α(Fe) solid solution, as reported by several authors [1,2,3].

In general, there is limited literature information available on hot-dip galvanising of steel grades with increased carbon content, let alone their behaviour during heat treatment. Jeon et al. [9] have reported in detail on the coating microstructure of a galvanised, high-Mn, medium-carbon steel coated at 450 °C/30 s. The coating was formed mainly by a thin layer of the δ phase and a comparatively thick layer of ζ underneath the η(Zn) top coating. The consequent annealing step at 550 °C/240 s was actually similar to a galvannealing step and caused the dissolution of the most of the η(Zn) into the ζ phase. Smirnov [17] summarised the behaviour of a wide range of steels with different carbon content during galvanising. Gogola et al. [18] reported more specifically on the phase evaluation of galvanised coatings on a high-carbon steel with the emphasis on the coating morphology. It was interesting to find a hot-dip coating microstructure with similar features between a high-carbon steel [18] and ductile iron castings with over 3.0 wt.% of C [19,20]. However, none of the studied literature sources investigated the behaviour of Zn-based coatings on medium-to-high-carbon steels after exposure to over 600 °C.

The current research aims to add information on the formation of hot-dip coatings on medium-carbon steels, including their behaviour during subsequent heat treatment. This includes galvannealing, which is aimed at modifying the coating microstructure, or even quenching and tempering, aimed at modifying the steel’s microstructure. In the cases of quenching (850 °C) and tempering (550 °C), the coating has to remain intact to limit extensive scale formation. On the other hand, the coating must not induce the formation of LME, which would deteriorate the mechanical properties of the heat-treated products.

## 2. Materials and Methods

The experimental samples were produced by hot-dip coating multiple 30 mm × 50 mm × 1.5 mm experimental steel sheet pieces. A standard steel for quenching and tempering with the designation C45 (1.0503) was used. Its chemical composition was measured on a spark-optical emission spectrometer (Q4 Tasman, Bruker, Billerica, MA, USA) and is shown in Table 1. The first set of samples was galvanised in a pure Zn bath, while the second bath consisted of Zn with 0.1 wt.% Al (Zn0.1Al). The 0.1 wt.% Al was chosen to limit the risk of LME during the prolonged heat exposure planned in the experiments [21].

**Figure 1:**
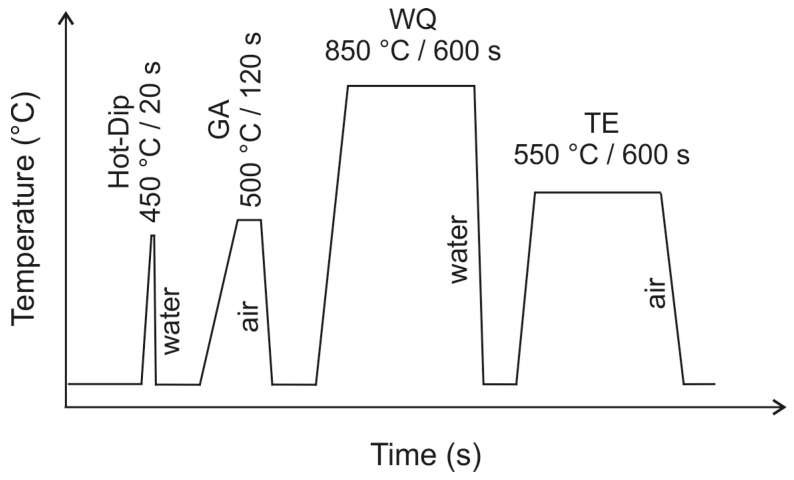
Schematic representation of the sample’s thermal history.

The steel sheet pieces were cleaned by pickling in a 20 vol.% aqueous solution of HCl acid for 60 s, followed by rinsing and dipping in an aqueous solution of 150 g ZnCl_2_.2NH_4_Cl flux in 1000 mL of demineralised water for about 30 s. The samples were removed from the flux bath and left to dry. The dry samples were dipped into the respective hot-dip baths. Hence, the samples were not preheated and a dwell time of 20 s was chosen for the hot-dip step. The samples were extracted from the hot-dip bath and immediately cooled in a room-temperature water bath. The hot-dip galvanising was followed by subsequent heat treatment steps as summarised in Figure 1, with the resulting sample designations listed in Table 2. The parameters for the quenching and tempering were selected based on the recommendation of the steel’s producer [22]. The temperature of 850 °C for 600 s was selected for the quenching step and 550 °C for 600 s was selected for the tempering. The actual temperature of the samples during all heat treatment steps was followed up via a K-type thermocouple attached directly to the reference sample.

Each of the produced samples was investigated using SEM/EDX and XRD. XRD was measured on the flat side of the samples, while SEM/EDX was performed on the cross-sections across the coating. The metallographic preparation involved grinding on a set of emery papers up to grit 4000 and polishing on diamond pastes down to a grain size of 0.25 µm. Ethanol was used for cooling and lubrication in all steps to avoid the unwanted corrosion of the investigated coatings. The final step was etching in a 0.2 vol.% Nital solution for 3 s.

The microstructure characterisation was carried out via a JEOL JSM 7600F scanning electron microscope (SEM, Jeol Ltd., Tokyo, Japan). The Schottky-type field emission electron source was operated at an accelerating voltage of 20 kV. A backscattered electron (BE) detector was used for the image acquisition.

The chemical composition measurements were carried out via an energy-dispersive X-ray spectrometer (Oxford Instruments plc, Abingdon, UK) with an Oxford Instruments X-Max silicon drift detector.

The XRD analysis was carried out by a PANalytical Empyrean X-ray diffractometer (XRD) (Malvern Panalytical Ltd., Malvern, UK). Bragg–Brentano geometry was utilised for the measurements. Theta-2Theta angle range was chosen to be between 20° and 145° 2Theta. The XRD source was set to 40 kV and 40 mA. The incident beam path was equipped with a 0.04 rad soller slit, 1/4° divergence slit and a 1/2° anti-scatter slit. The diffracted beam path consisted of a 1/2° anti-scatter slit, 0.04 rad soller slit, Fe beta filter and PIXcel3D position-sensitive detector operated in 1D scanning mode. The phase quality was evaluated using the PANalytical Xpert High Score program (HighScore Plus version 3.0.5) with the ICSD FIZ Karlsruhe database. Quantitative results were calculated from the XRD patterns using the program MAUD version 2.84 [23]. The program used Rietveld refinement based on an asymmetric pseudo-Voight function to describe experimental peaks. An anisotropy size-strain model was applied to describe the FWHM of the peaks. The microstructure was occasionally heavily textured, resulting in significant discrepancy between the nominal and measured peak intensities. This was corrected using the spherical harmonic functions with fibre symmetry. The quality of the fit was targeted to be below 15% R_wp_.

As the measurements were performed directly on the sample surface after each production step, oxides were also measured on the surface. Their presence significantly influenced the overall quantitative results; hence, only relative volume fractions were reported.

## 3. Results

The first objective of our research was to confirm that the prepared coating remains compacted enough throughout all production steps to protect the steel substrate from unwanted oxidation. Additionally, no signs of LME were to be observed. Both could be observed by investigating the cross-sections of all samples.

On the other hand, XRD measurements of the sample surfaces could provide information about the phase composition of a statistically larger surface area.

### 3.1. SEM/EDX Analysis of Cross-Sections

For easier reference, cross-section investigation was divided into chapters based on the individual production steps, while comparing the Zn and Zn0.1Al coatings side by side.

Backscattered-electron scanning electron microscopy (BSEM) images of all the investigated samples are shown in the following chapters. The phases observed via SEM/EDX in all studied samples were classified based on their chemical composition. The range of chemical composition for each phase considered is given in Table 3 [1,5,6,24].

#### 3.1.1. Hot-Dip Coating

**Figure 2:**
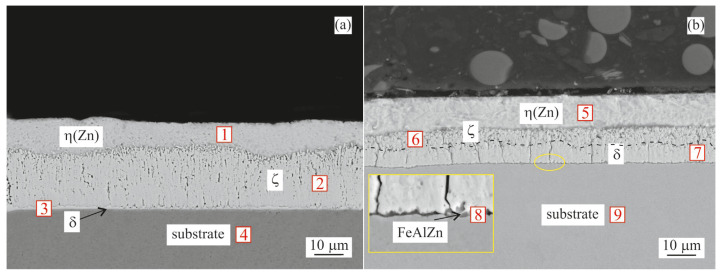
BSEM cross-section images of the samples after hot dip: (**a**) Zn coating; (**b**) Zn0.1Al coating. Points 1–9 indicate point measurement sites for the EDX analysis summarized in Table 4.

Figure 2 shows the typical cross-section microstructure of the hot-dip-coated samples, including multiple EDX measurement sites. Measured values are listed in Table 4.

The coating formed in a pure Zn bath shown in Figure 2a is formed by the δ layer at the substrate, followed by the ζ and η(Zn) layers. The intermetallic layer, including all possible intermetallic phases, reached an average thickness of ~20 µm, while the η(Zn) layer reached ~10 µm.

Figure 2b shows the cross-section of the coating formed in a Zn0.1Al bath. A very thin (<1 µm) FeAlZn interfacial layer was formed directly on the substrate. As listed in Table 4, an increased amount of Al was indicated in site No. 8. The exact composition of this layer could not be directly measured via SEM/EDX due to its low thickness; hence, we relied on literature input to confirm its presence [6,14]. The δ and ζ layers were visible above this FeAlZn layer, followed by the η(Zn) layer. The thickness of the overall intermetallic layer was ~12 µm with ~10 µm of η(Zn) on the top.

#### 3.1.2. Galvannealing

After the galvannealing step for both coating compositions, the η(Zn) layer dissolved, decreasing the total coating thickness, while increasing the thickness of the intermetallic layer. This was also confirmed by the corresponding EDX analysis (Table 5).

**Figure 3:**
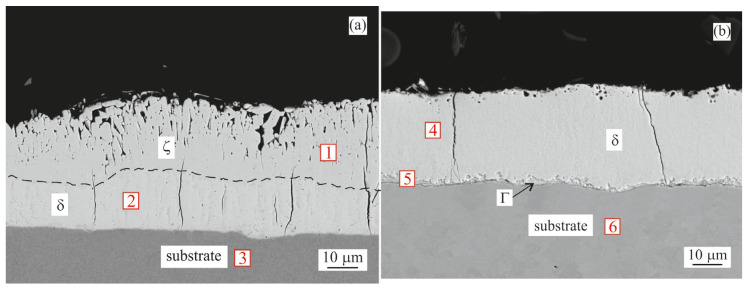
BSEM cross-section images of the samples after galvannealing: (**a**) Zn coating; (**b**) Zn0.1Al coating. Points 1–6 indicate point measurement sites for the EDX analysis summarized in Table 5.

In the Zn–GA sample both the ζ and δ layers increased in thickness, as shown in Figure 3a. The overall thickness of the intermetallic layer increased to ~40 µm.

In case of the Zn0.1Al–GA sample, the intermetallic layer was formed by a thin layer of Γ phase and a ~30 µm thick layer of δ phase.

**Table 5:** Chemical composition (EDX) of measurement sites from Figure 3.

Coating	Site No.	Fe Content [at.%]	Zn Content [at.%]	Phase
	1	6	94	ζ
Zn	2	10	90	δ
	3	100	-	Substrate
	4	11	89	δ
Zn0.1Al	5	31	69	Γ
	6	100	-	Substrate

#### 3.1.3. Water Quenching

**Figure 4:**
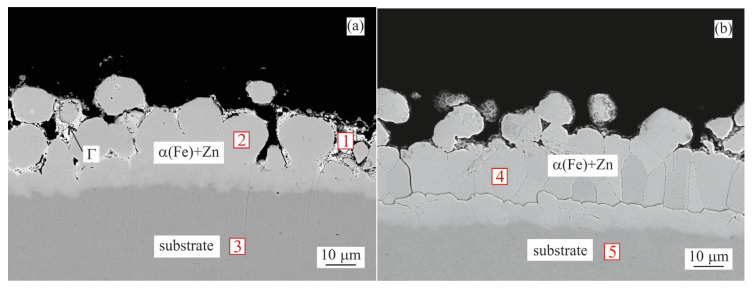
BSEM cross-section images of the samples after water quenching: (**a**) Zn coating; (**b**) Zn0.1Al coating. Points 1–5 indicate point measurement sites for the EDX analysis summarized in Table 6.

Due to the high heat-treatment temperature (850 °C) and subsequent rapid cooling to room temperature (water quenching), a supersaturated α(Fe)-based solid solution with increased Zn content was formed. In the images, it is designated as α(Fe) + Zn. This solid solution formed most of the coating in both cases (Figure 4a,b). Only for the Zn coating (Figure 4a) was the Γ phase observed along the supersaturated α(Fe) s.s. This was also confirmed by EDX analysis and summarised in Table 6. The coating remained intact even after this heat treatment step, but thickness value could only be estimated due to the high roughness of the coating surface. For the Zn coating it was ~20–25 µm, while for the Zn0.1Al it was ~25–30 µm.

#### 3.1.4. Tempering

**Figure 5:**
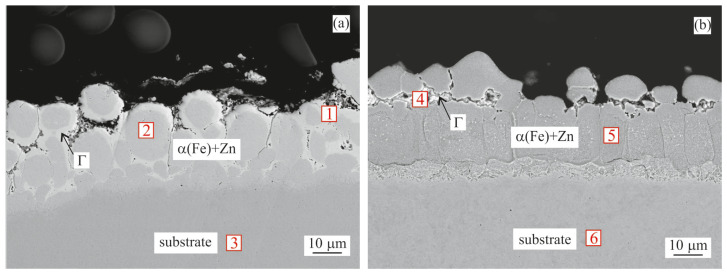
BSEM cross-section images of the samples after tempering: (**a**) Zn coating; (**b**) Zn0.1Al coating. Points 1–6 indicate point measurement sites for the EDX analysis summarized in Table 7.

During the tempering step, the breakdown of the supersaturated α(Fe) + Zn occurred. As seen in Figure 5a, for the Zn coating this was carried out by creating the Γ phase around the α(Fe) + Zn particles. For the Zn0.1Al coating, a similar phenomenon could be observed, but on a smaller scale (Figure 5b). Table 7 shows that the Zn content of the α(Fe) + Zn was slightly lower compared to the values in Table 6. Again, due to the high roughness of the coating surface, the thickness values could only be estimated. For the Zn coating it was ~25 µm, while for the Zn0.1Al it was on the level of ~30 µm.

### 3.2. XRD Analysis of the Surface

**Figure 6:**
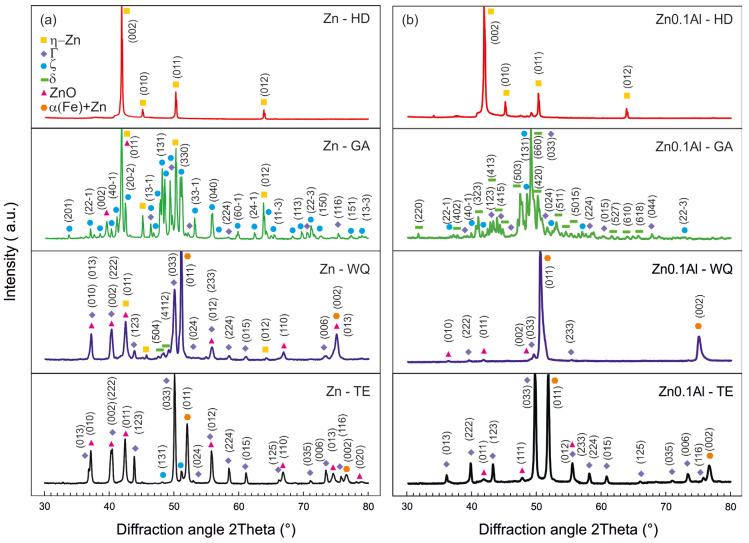
XRD patterns recorded on the sample surface after each processing step: (**a**) Zn coating; (**b**) Zn0.1Al coating.

XRD measurements were performed on the sample surface after each processing step. Table 8 contains the list of phases identified (ICSD FIZ Karlsruhe database) and confirmed via Rietveld refinement. All diffraction patterns are summarised in Figure 6.

Patterns for the Zn-coated samples are compared in Figure 6a. Relative quantities of the detected phases are summarised in Table 9. The surface of the galvanised sample (Zn–HD) was formed only by the η(Zn) phase. As expected, this phase was shown to be heavily textured, resulting in significant discrepancy between the nominal and measured peak intensities. The galvannealing step mainly enabled the formation of the ζ phase by transforming the η(Zn) phase. Γ was also marginally identified. During the exposure to 850 °C temperature, a significant amount of Zn from other intermetallic phases was dissolved in an α(Fe)-based solid solution. The subsequent rapid quenching into water caused the formation of the Fe-Zn supersaturated solid solution. Additionally, Γ and δ were also confirmed on the surface. During tempering at 550 °C, the α(Fe) + Zn partly decomposed, mainly forming the Γ phase along with a minor portion of the ζ phase.

In most patterns, Zn-based oxides were also identified on the surface.

Figure 6b contains all XRD patterns recorded for the Zn0.1Al-coated samples, while relative phase quantities are listed in Table 10. Similar to the Zn–HD sample, this coating also perfectly covered the surface with a layer of η(Zn) phase. After galvannealing the surface was formed mainly by the δ phase with a smaller portion of Γ and ζ phases. Additionally, for the Zn0.1Al coating, the supersaturated α(Fe) + Zn phase was mainly formed after the water quenching. Only a tiny portion of the Γ phase was identified. The tempering step once again caused mainly the decomposition of α(Fe) + Zn to the Γ phase.

## 4. Discussion

After galvanising (450 °C/20 s), a standard hot-dip coating was formed by the commonly known intermetallic layers (ζ and δ) underneath the η(Zn) layer, as confirmed by SEM/EDX (Figure 2). These were similar for both types of coatings tested (Zn vs. Zn0.1Al). The Zn0.1Al coating formed a thinner total intermetallic layer with about 12 µm compared to 20 µm for the pure Zn coating (Figure 2a,b), while the δ phase seemed to be more preferred in the Zn0.1Al coating. This was most probably caused by the formation of a discontinuous FeAlZn-based inhibition layer formed due to the Al in the Zn0.1Al hot-dip bath. Baril et al. [14] confirmed the presence of these FeAlZn-based phases for baths with 0.10–0.13 wt.% Al. Price et al. [13] reported that ~0.1 wt.% Al supported the formation of a FeAl_2_Zn_x_ phase. However, Zapico-Álvarez et al. [15] identified this non-uniform layer in a 0.11 wt.% Al coating to be built by a ~20 nm thick Fe_2_Al_5_-Zn_x_ phase followed by a δ phase layer. As reported by several authors, 0.1 wt.% Al is the borderline composition which started to act in favour of the δ phase instead of ζ phase formation [13,15,21,25,26]. The top coating was formed for both coatings by a uniform layer of η(Zn) of about 10 µm. As a result, the XRD measurement identified only the presence of the η(Zn) phase (Figure 6a,b) on the top surface of the HD samples.

As a consequence of galvannealing (500 °C/120 s), the η(Zn) layer was almost completely dissolved, increasing the overall intermetallic layer thickness. In the case of the Zn coating, the ζ and δ layers grew to an overall thickness of ~40 µm (Figure 3a). As the ζ layer was on top, the XRD measurements identified it as the dominant phase with over 60 vol.% along with a portion of the undissolved η(Zn) phase (Figure 6a, Zn–GA). On the other hand, in the Zn0.1Al coating, the δ phase was predominantly formed (Figure 3b). Overall intermetallic layer thickness was ~30 µm. XRD confirmed that the surface was formed mainly by the δ phase, almost 80 vol.%, along with a small amount of the Γ phase (Figure 6b, Zn0.1Al GA). Similar behaviour was observed by other literature sources [6,7,8,9].

The first step aimed specifically at the heat treatment of the substrate was quenching. The austenitisation temperature of 850 °C was in the field of the Zn-rich α(Fe) solid solution [5]. The fast water quenching from this temperature caused the retention of the Zn-rich supersaturated α(Fe) s.s. Similarly, other authors [1,2,3,27] also observed the formation of the supersaturated α(Fe) s.s. after annealing hot-dip-coated substrates at temperatures between 600–900 °C and subsequent fast cooling at ~30 °C/s. For the Zn-coated samples, over 40 vol.% of the surface was formed by this solid solution, but δ and Γ phases were also created with up to 20 vol.% each (Figure 6a, Zn–WQ). The Zn0.1Al coating contained almost exclusively the supersaturated α(Fe) solid solution with over 95 vol.% on the top surface (Figure 6b, Zn0.1Al–WQ). For both coatings, the chemical composition of the supersaturated α(Fe) was measured by SEM/EDX, showing a Zn content of over 37 at.% (Table 6) which is again in line with the literature sources [1,2,3]. After the WQ step, both coatings were still intact as viewed via SEM, but the surface roughness increased significantly. Average coating thicknesses were comparable between 20 and 30 µm (Figure 4a,b).

Tempering, to reduce the steel’s internal stresses, was the final heat treatment step (550 °C/600 s). At this temperature, the supersaturated α(Fe) s.s. reduced its Zn content by the diffusion. SEM/EDX indicated a Zn content of these areas to be lower by 6–13 at.% compared to the WQ state (Table 7). The Zn expelled from the supersaturated α(Fe) s.s. was mainly utilised in both coatings to form particles of the Γ phase (Figure 5a,b). For the Zn coating, the XRD measurements indicated over 40 vol.% of Γ phase with less than 20 vol.% of supersaturated α(Fe) s.s. remaining (Figure 6a). The Zn0.1Al coating showed a similar trend, with over 45 vol.% of both Γ and supersaturated α(Fe) s.s. phases being identified (Figure 6b). Comparing the peak positions for supersaturated α(Fe) s.s. between WQ and TE states, a discrepancy was clearly observed. This was caused by the reduction of the supersaturated α(Fe) s.s. lattice parameter as a consequence of reducing its Zn content. This trend was also indicated by literature sources [28,29,30]. The morphology of the Γ phase was different between these two coatings as observed on the SEM images (Figure 5a,b). In the Zn coating, it was formed mainly along the supersaturated α(Fe) s.s. grain boundaries, while in the Zn0.1Al coating tiny particles were also observed within the supersaturated α(Fe) s.s. grains. This phenomenon was probably caused by the difference in the coating morphology. The supersaturated α(Fe) s.s. grains in the Zn0.1Al coating were more homogeneous and closer-packed, while in the Zn coating, they were larger and less closely arranged. The thickness of the intact coating was between 25–30 µm for both coatings.

Liquid metal embrittlement (LME) was a phenomenon that was followed up after each process step. However, after detailed analysis of the substrate, microstructures were not observed in any case. This confirmed that such an approach was also feasible for steels with increased carbon content processed by quenching and tempering.

## 5. Conclusions

The aim of this research was to confirm the feasibility of applying a Zn-based coating to limit the scale formation during the heat treatment of medium-carbon steels. A pure Zn coating and a Zn0.1Al coating were tested.

- The Zn0.1Al coating resulted in a slightly more compact final coating, but both coatings performed similarly enough to be applicable.
- The experimental results confirmed that a sufficient Zn-based coating remained on the steel surface after each of the processing steps even after exposure to 850 °C. In all cases, at least a 20 µm thick, intact coating remained after all processing steps.
- The coatings had their specific phase composition after each heat treatment step. η(Zn), ζ and δ were formed after galvanising. η(Zn) was transformed during galvannealing mainly into ζ, δ and Γ phases.
- After the water quenching, a supersaturated α(Fe)-based s.s. was formed. This phase was usually not observed in relation to hot-dip coatings, and it was initiated by the specific conditions of water quenching from 850 °C.
- During tempering (550 °C/600 s), a significant portion of the supersaturated α(Fe) s.s. was transformed mainly to the Γ phase. This did not, however, impede the protective properties of the coating regarding the limitation of iron scale formation.
- LME phenomena were not observed after any of the process steps.
- It is feasible to use this approach to control the steel’s scale formation during quenching and tempering.
- This enables smaller-scale production facilities to carry out forming on steel sheets and subsequently carry out the heat treatment (quenching and tempering) inhouse without the risk of heavy iron scale formation.

## Figures and Tables

**Table 1:** Chemical composition of the steel substrate (1.0503) used in the experiments.

Element	C	Si	Mn	Cr	Mo	Ni	Cu	Fe
Content in wt.%	0.506	0.249	0.567	0.304	0.075	0.200	0.344	bal.

**Table 2:** Designation of experimental steps and investigated sample.

Production Step	1. Hot-Dip Galvanising (Hot-Dip)	2. Galvannealing (GA)	3. Water Quenching (WQ)	4. Tempering (TE)
Designations of Zn-coated samples	Zn–HD	Zn–GA	Zn–WQ	Zn–TE
Designations of Zn0.1Al-coated samples	Zn0.1Al–HD	Zn0.1Al–GA	Zn0.1Al–WQ	Zn0.1Al–TE

**Table 3:** List of phases considered by SEM/EDX based on their chemical composition [1,5,6,24].

Phase Name	Symbol/Designation	Fe Content [at.%]	Zn Content [at.%]
Substrate	α(Fe)	99–100	0–1
Supersaturated α(Fe) solid solution	α(Fe) + Zn	58–98	2–42
Gamma	Γ	19–31	69–81
Delta	δ	8–13	87–92
Zeta	ζ	6–7	93–94
Eta Zn solid solution	η(Zn)	0–0.04	99.96–100

**Table 4:** Chemical composition (EDX) of measurement sites from Figure 2.

Coating	Site No.	Fe Content [at.%]	Zn Content [at.%]	Al Content [at.%]	Phase
Zn	1	0.5	99.5	-	η(Zn)
	2	7	93	-	ζ
	3	12	88	-	δ
	4	100	-	-	Substrate
Zn0.1Al	5	1	99	-	η(Zn)
	6	7.5	92.5	-	ζ
	7	12	88	-	δ
	8	50	47	3	FeAlZn interfacial layer
	9	100	-	-	Substrate

**Table 6:** Chemical composition (EDX) of measurement sites from Figure 4.

Coating	Site No.	Fe Content [at.%]	Zn Content [at.%]	Phase
	1	26	74	Γ
Zn	2	62	38	α(Fe) + Zn
	3	100	-	Substrate
Zn0.1Al	4	63	37	α(Fe) + Zn
	5	100	-	Substrate

**Table 7:** Chemical composition (EDX) of measurement sites from Figure 5.

Coating	Site No.	Fe Content [at.%]	Zn Content [at.%]	Phase
	1	30	70	Γ
Zn	2	69	31	α(Fe) + Zn
	3	100	-	Substrate
	4	32	68	Γ
Zn0.1Al	5	77	23	α(Fe) + Zn
	6	100	-	Substrate

**Table 8:** List of phases identified via XRD.

Phase Name	Symbol/Designation	Phase Chemical Formula	Crystal System	Space Group Number	ICSD Database Number
Fe-Zn solid solution	α(Fe) + Zn	Fe_0.75_Zn_0.25_	bcc	229	01-080-4455
Gamma	Γ	Fe_4_Zn_9_	bcc	217	03-065-4386
Delta	δ	Fe_13_Zn_126_	hexagonal	194	01-083-4808
Zeta	ζ	FeZn_13_	monoclinic	12	98-016-3222
Eta Zn solid solution	η(Zn)	Zn	hcp	194	98-065-3502
ZnO	ZnO	ZnO	hexagonal	186	01-078-4606

**Table 9:** Relative volume fractions identified during XRD analysis of Zn-coated samples.

Production Step	η(Zn)	ζ	δ	Γ	α(Fe) + Zn	ZnO
Zn–HD	■■■■	--	--	--	--	--
Zn–GA	■■■	■■■■	--	■	--	■
Zn–WQ	■	■	■■	■■■	■■■■	■■
Zn–TE	--	■■	--	■■■■	■■	■■■

Legend: ■■■■: dominant phase; ■■■: significant portion; ■■: lower relative amount; ■: low degree of certainty; --: not detected.

**Table 10:** Relative volume fractions identified during XRD analysis of Zn0.1Al-coated samples.

Production Step	η(Zn)	ζ	δ	Γ	α(Fe) + Zn	ZnO
Zn0.1Al–HD	■■■■	--	--	--	--	■
Zn0.1Al–GA	--	■	■■■■	■■	--	--
Zn0.1Al–WQ	--	--	--	■	■■■■	■
Zn0.1Al–TE	--	--	--	■■■	■■■	■

Legend: ■■■■: dominant phase; ■■■: significant portion; ■■: lower relative amount; ■: low degree of certainty; --: not detected.

## Data Availability

Data sharing is not applicable.
